# High Parity Predicts Poor Outcomes in Patients With Luminal B-Like (HER2 Negative) Early Breast Cancer: A Prospective Finnish Single-Center Study

**DOI:** 10.3389/fonc.2020.01470

**Published:** 2020-08-14

**Authors:** Anniina Jääskeläinen, Nelli Roininen, Peeter Karihtala, Arja Jukkola

**Affiliations:** ^1^Department of Oncology and Radiotherapy, Medical Research Center Oulu, Oulu University Hospital and University of Oulu, Oulu, Finland; ^2^Department of Oncology, Helsinki University Hospital Comprehensive Cancer Centre, University of Helsinki, Helsinki, Finland; ^3^Department of Oncology and Radiotherapy, Tampere University Hospital, Tampere, Finland; ^4^Faculty of Medicine and Health Technology, Tampere University, Tampere, Finland; ^5^Tampere Cancer Center, Tampere, Finland

**Keywords:** breast carcinoma, deliveries, parity, prognosis, subtypes, survival

## Abstract

While breast cancer prognoses are generally good, different molecular subtypes are known to have varying outcomes. Previous studies using breast cancer registries have suggested that high parity may be an adverse prognostic factor in luminal breast cancer, but breast cancer subtype definitions have varied and there have been few prospective studies. We therefore collected prospective data from patients diagnosed with early breast cancer at a single institution and followed them for a median of 8.5 years. All patients (*N* = 594) were treated according to Finnish national guidelines using modern treatment modalities in a Finnish university hospital. Clinicopathological surrogates of the intrinsic breast cancer subtypes were updated to match European Society for Medical Oncology 2015 Early Breast Cancer Clinical Practice Guidelines. The overall 10-year breast cancer–specific survival (BCSS) was 91.4%, with the longest 10-year BCSS observed in luminal A-like cancers (97.9%) and the worst in luminal B-like (HER2 positive) cancers (80.6%). Parity of ≥ 5 deliveries was also associated with poor BCSS (univariate *P* = 0.0020). However, when the subtypes were assessed separately in a multivariate analysis that included tumor size and nodal status, high parity remained significant only in luminal B-like (HER2 negative) cancers (HR = 2.63; 95% confidence interval = 1.04–6.62; *P* = 0.040). Our results suggest excellent overall 10-year BCSS but indicate that high parity is an adverse prognostic factor in luminal B-like (HER2 negative) breast cancers.

## Introduction

Various gynecological and reproductive patient history factors have been associated with increased breast cancer risks and prognoses (1, 2). Complex reproductive factors continue to be significant determinants of breast cancer risk: breast cancer risks seems to be transiently higher during the initial years after delivery, but are subsequently reduced for a prolonged period of time (3, 4). It has been suggested that the initial risk increase risk may be due to hormonal stimuli leading to proinflammatory activation and changes in tumor microenvironments (5, 6).

Previous findings have also suggested that pregnancy-related effects on breast cancer incidence and prognoses may vary among breast cancer subtypes (7–9). The five intrinsic breast cancer subtypes, which are classed according to their immunohistochemical (IHC) surrogates using the European Society for Medical Oncology (ESMO) 2015 Early Breast Cancer Clinical Practice Guidelines, differ in terms of both their molecular constitutions and patient prognoses (10–12). Although evidence about the prognostic role of patient reproductive history is still emerging, it has become clear that reproduction-related risk factors differ among the breast cancer subtypes (13–15). In particular, registry- and population-based studies have suggested that high parity is an adverse prognostic factor in luminal subtypes and in triple-negative breast cancer (TNBC); however, breast cancer subtype definition has varied, and there have been few prospective studies (7, 9, 13, 15, 16).

Moreover, although breast cancer survival and mortality have been well studied (17, 18), there have been few prospective studies that report on long-term breast cancer–specific survival (BCSS) for each molecular subtype of breast cancer using real-world data. The purpose of the present study was therefore to evaluate subtype-specific long-term outcomes and the importance of reproductive anamnesis as potential prognostic factors in patients diagnosed with early breast cancer.

## Materials and Methods

Prospective patient data were collected at Oulu University Hospital in 2003–2013. All participants (*N* = 594) had been diagnosed with early, invasive breast cancer and received treatment at Oulu University Hospital (Table 1). Patients with previous breast cancer diagnoses or distant metastases at the time of diagnosis were excluded.

**Table 1:** The distribution of patient characteristics.

**Age at breast cancer onset**	
28–40 years	37 (6.2%)
41–74 years	511 (86.0%)
75–87 years	46 (7.7%)
Median	58 years
**Age at menarche**	
10–12 years	159 (26.8%)
13–14 years	205 (34.5%)
15–17 years	97 (16.3%)
Missing	133 (22.4%)
Median	13 years
**Menopausal status**	
Premenopausal	156 (26.3%)
Perimenopausal	10 (1.7%)
Postmenopausal	390 (65.7%)
Missing	38 (6.4%)
Median age at menopause	48 years
**Number of deliveries before breast cancer diagnosis**	
0	67 (11.3%)
1	77 (13.0%)
2	186 (31.3%)
3	118 (19.9%)
4	45 (7.6%)
≥ 5	41 (6.9%)
Missing	60 (10.1%)
Median number of deliveries	2

The assessments of these prognostic factors were determined at time of the initial diagnosis in the accredited Oulu University Hospital pathology laboratory as a part of routine diagnostics. Based on this, tumors were classed into five intrinsic subtypes according to ESMO Early Breast Cancer Clinical Practice Guidelines (12). Luminal A-like carcinomas expressed both estrogen receptors (ER) and progesterone receptors (PR), but HER2 was not overexpressed and Ki-67 was expressed in <15% of their cells. Luminal B-like (HER2 negative) carcinomas were also ER positive and HER2 negative, but they either showed Ki-67 expression in >15% of their cells or were PR negative. Luminal B-like (HER2 positive) carcinomas still expressed ER, but they also overexpressed HER2. TNBCs were defined as tumors with no expression of ER, PR and HER2. HER2 positive (non-luminal) cases overexpressed HER2 but did not express either ER or PR. Table 2 details the subtypes present in our study cohort.

**Table 2:** The distribution of tumor characteristics.

**T class**	
T1	384 (64.6%)
T2	189 (31.8%)
T3	19 (3.2%)
T4	2 (0.3%)
**N class**	
N0	368 (62.0%)
N1	162 (27.3%)
N2	50 (8.4%)
N3	14 (2.4%)
**Histopathology**	
Ductal	456 (76.8%)
Lobular	91 (15.3%)
Other	47 (7.9%)
**Histopathological grade**	
Grade 1	103 (17.3%)
Grade 2	293 (49.3%)
Grade 3	172 (29.0%)
Unknown	26 (4.4%)
**ER expression**	
Negative (0%)	92 (15.5%)
Weak (1–9%)	18 (3.0%)
Moderate (10–59%)	26 (4.4%)
High (> 59%)	455 (76.6%)
Unknown	3 (0.5%)
**PR expression**	
Negative (0%)	146 (24.6%)
Weak (1–9%)	81 (13.6%)
Moderate (10–59%)	65 (10.9%)
High (> 59%)	298 (50.2%)
Unknown	4 (0.7%)
**HER2 status**	
HER2 positive (CISH)	61 (10.3%)
HER2 negative	533 (89.7%)
**Ki-67 expression**	
Negative (<5%)	41 (6.9%)
Weak (5–14%)	268 (45.1%)
Moderate (15–30%)	141 (23.7%)
High (> 30%)	136 (22.9%)
Variable status or unknown	8 (1.4%)
**Tumor type**	
Unifocal	472 (79.5%)
Multifocal	122 (20.5%)
**Breast cancer subtypes**	
Luminal A-like	271 (45.6%)
Luminal B-like (HER2 negative)	192 (32.3%)
Luminal B-like (HER2 positive)	33 (5.6%)
HER2 positive, non-luminal	27 (4.5%)
Triple negative	63 (10.6%)
Unknown	8 (1.4%)
**Subtype division in patients with five or more deliveries**	
Luminal A-like	17 (41.5%)
Luminal B-like (HER2 negative)	16 (39.0%)
Luminal B-like (HER2 positive)	2 (4.9%)
HER2 positive, non-luminal	0
Triple negative	5 (12.2%)
Unknown	1 (2.4%)

*ER, estrogen receptor; PR, progesterone receptor*.

Histopathology was evaluated according to current WHO classifications, and tumor stage was assessed according to TNM classifications (19). The expressions of ER, PR and Ki-67 were assessed using the IHC methods previously described (20). HER2 expression was then assessed using IHC and chromogenic *in situ* hybridization (CISH) to confirm any positive results. Any sample with a positive result of six or more gene copies according to CISH was considered to be HER2 positive (21).

### Statistical Analyses

Statistical analyses were performed using SPSS Statistics software version 25.0 for Mac (IBM Corporation, Armonk, NY, USA). Survival was analyzed using Kaplan-Meier curves and the log-rank test. BCSS was calculated from the date of surgical tumor removal to the time of breast cancer–related death. Prognostic factors were reformatted as two-classed variables for the analyses. The effect of parity in survival was assessed using five deliveries as a cut-off point. Multivariate analyses were conducted using Cox multivariate regression analyses (the co-variates were tumor size and nodal status); tumor sizes were assessed as either T1 or T2–4 and nodal status was categorized as either N0 or N1–3. Crosstabulation was used to compare the groups and two-sided Pearson's Chi-square test or Fisher's exact test were used as applicable to determine significance. Continuous variables were assessed using the Mann Whitney *U* test or Pearson Correlation test. *P*-values < 0.05 were considered significant.

## Results

### Clinical Characteristics

Median follow-up time was 102 months (range 2–186). During follow-up, 34 patients (5.7%) were diagnosed with local relapse and 60 patients (10.1%) were diagnosed with distant metastases. The most frequent single site of metastasis was in the bones (17 patients, 2.9%), but multiple-site metastasis was most frequent (24 patients, 4.0%). 88 (14.8%) of the patients had ER- and PR- phenotype in their tumors, 58 (9.8%) had ER+ and PR-, 4 (0.7%) ER– and PR + and 444 (74.7%) phenotype, respectively. Altogether 347 (58.4%) of the patients received adjuvant chemotherapy (Table 3). The frequency of adjuvant chemotherapy was the lowest within the patients with luminal A subtype (97 patients, 35.8%). 130 (67.7%) of the luminal B-like (HER2 negative) patients received adjuvant chemotherapy and the frequency was even higher with the patients with HER2 overexpression subtype (27 patients, 100%), luminal B-like (HER2 positive) subtype (30 patients, 90.9%) and triple-negative subtype (57 patients, 90.5%). Subtype distribution (Table 2) varied among the age groups. In patients ≤ 40 years, the most frequent subtype was TNBC, while in both of the older age groups (41–74 and ≥ 75), the most frequent subtype was the luminal A-like subtype (Table 4). Parity was recorded at the initial time of the early breast cancer diagnosis. Although the median number was 2 (range 0–12), 41 patients had ≥ 5 deliveries. The highest percentage of nulliparous women (18.5%) was observed among patients with HER2 positive (non-luminal) breast cancer. These results are presented in detail in Table 1.

**Table 3:** The distribution of adjuvant chemotherapy, radiotherapy, and endocrine therapy.

**Adjuvant chemotherapy**	
Anthracycline-based + taxane	129 (21.8%)
Anthracycline-based	130 (21.9%)
Other chemotherapy	25 (4.2%)
Trastuzumab + chemotherapy	61 (10.3%)
No adjuvant chemotherapy	247 (41.6%)
Missing	2 (0.3%)
**Adjuvant radiation therapy**	
Yes	514 (86.5%)
No	80 (13.5%)
**The first prescribed adjuvant endocrine therapy**	
Tamoxifen	164 (27.6%)
Aromatase inhibitor	222 (37.4%)
Tamoxifen + goserelin	2 (0.3%)
Other endocrine therapy	3 (0.5%)
No endocrine therapy	200 (33.7%)
Missing	3 (0.5%)

**Table 4:** Subtype distribution for each age group.

**Breast cancer subtypes according to age**	**594 (100%)**
28–40 years	37 (6.2%)
Luminal A-like	9 (24.3%)
Luminal B-like (HER2 negative)	11 (29.7%)
Luminal B-like (HER2 positive)	1 (2.7 %)
HER2 positive, non-luminal	3 (8.1%)
Triple negative	13 (35.1%)
Unknown	0
41–74 years	511 (86.0%)
Luminal A-like	238 (46.6%)
Luminal B-like (HER2 negative)	168 (32.9%)
Luminal B-like (HER2 positive)	30 (5.9%)
HER2 positive, non-luminal	24 (4.7%)
Triple negative	45 (8.8%)
Not fitting any subtype	1 (0.2%)
Unknown	5 (1.0%)

### Survival and Parity

**Figure 1:**
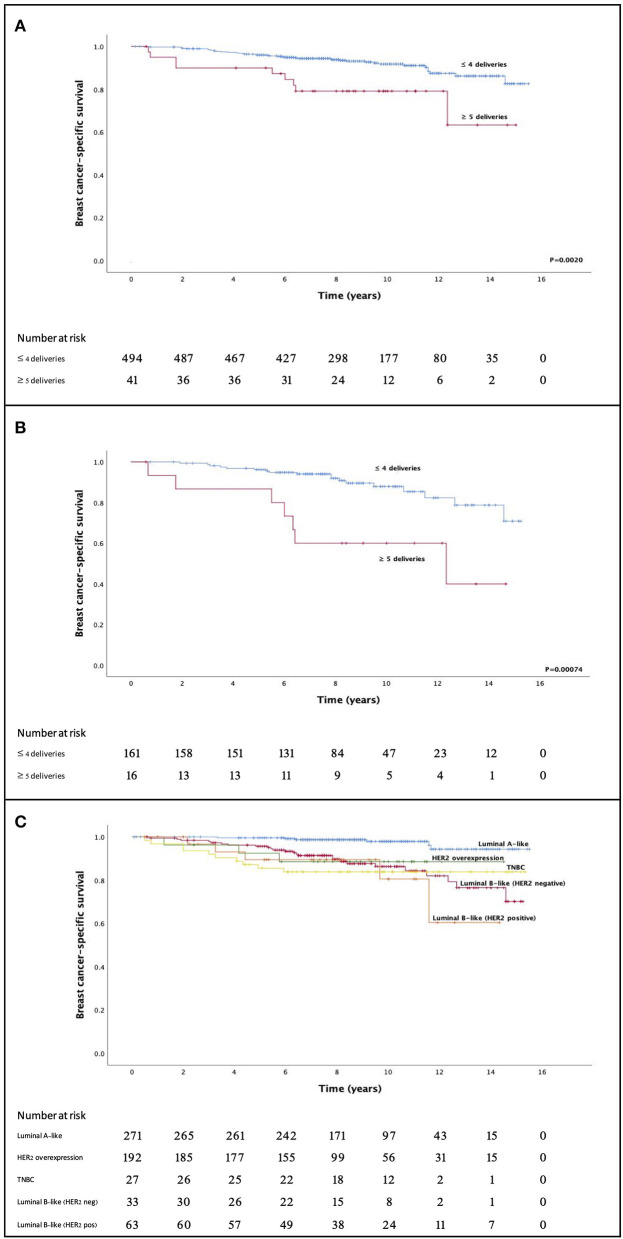
High parity (≥5 deliveries) before breast cancer diagnosis predicted poor breast cancer-specific survival in the overall cohort **(A)** and separately in luminal B-like (HER2 negative) breast cancers **(B)**. **(C)** Shows the long-term breast cancer-specific survival according to the intrinsic subtype immunohistochemical surrogates. TNBC, triple-negative breast cancer.

Having ≥5 deliveries was the optimal cut-off as the predictor of BCSS. Overall, having ≥5 deliveries was associated with a poor BCSS (*P* = 0.0020) in univariate analyses; however, this result was not confirmed in multivariate analyses (Figure 1). When the subtypes were assessed separately high parity was significantly correlated with poor BCSS only in luminal B-like (HER2 negative) cancers (log-rank *P* = 0.00074). This was determined using a multivariate analysis (HR = 2.63; 95% CI = 1.04–6.62; *P* = 0.040) that included tumor size (T1 vs. T2–4; HR = 2.98; 95% CI = 1.27–6.99; *P* = 0.012) and nodal status (N0 vs. N1–3; HR = 5.21; 95% CI = 1.70–16.0; *P* = 0.0039) (Table 5). When age at breast cancer onset (a continuous variable) was included to the Cox regression model along with parity, having ≥5 deliveries was still an independent prognostic factor with the HR of 3.45 (95% CI 1.62–7.38; *P* = 0.001), while age was not significant with the HR of 0.98 (95% CI 0.96–1.01; *P* = 0.22). There was no difference in BCSS when parous and nulliparous women were compared (*P* = 0.991).

**Table 5:** 5- and 10-year breast cancer–specific survival (BCSS).

**Subtype**	**5-year BCSS**	**10-year BCSS**
Luminal A-like	99.6%	97.9%
HER2 overexpression	92.4%	88.6%
Luminal B-like (HER2 negative)	95.7%	86.3%
TNBC	85.6%	83.9%
Luminal B-like (HER2 positive)	89.5%	80.6%
Whole cohort	95.8%	91.4%

### Associations Between Deliveries and Clinicopathological Characteristics

High parity (≥5 deliveries) before an initial breast cancer diagnosis was associated with the presence lymph node metastases in a whole-cohort analysis (*P* = 0.0020), but not when the subtypes were assessed separately. Overall, 24.4% of patients with ≥ 5 deliveries had a distant recurrence during the follow-up period, in comparison to 9.91% in the group with ≤ 4 deliveries (*P* = 0.0090). The number of deliveries did not associate with T-class, grade, Ki-67 expression, subtype, HER2, ER or PR expression or the presence of bilateral or multifocal breast cancer. The absolute number of deliveries correlated with the age of breast cancer onset (*P* = 0.000078; correlation co-efficient 0.170).

### Survival and Subtypes

Overall, the estimated 5- and 10-year BCSS were 95.8 and 91.4%, respectively (Table 6). The longest estimated 5- and 10-year BCSS were observed in luminal A-like cancers (99.6 and 97.9%, respectively), while the worst 5-year BCSS was observed in the TNBC subgroup (85.6%) and the worst 10-year BCSS was seen in the luminal B-like (HER2 positive) subgroup (80.6%). When the subtypes' BCSS were compared, the luminal A-like subtype had a more favorable outcome than any other subgroup (*P* < 0.005 for all). We did not observe any other statistically significant differences in BCSS between the subgroups.

**Table 6:** Having five or more deliveries at the time of breast cancer diagnosis is indicator of poor prognosis in multivariate analysis.

		**95% confidence interval**		***p*-value**
	**Hazard ratio**	**Lower**	**Upper**	
≥ 5 deliveries	2.628	1.043	6.620	0.040
T2-4	2.983	1.273	6.993	0.012
N1-3	5.208	1.696	15.989	0.0039

## Discussion

We collected and analyzed prospective data from 594 patients with invasive early breast cancer. Using IHC surrogates, the tumors were divided into five molecular subtype groups according to the ESMO Early Breast Cancer Clinical Practice Guidelines. Notably, we observed that high parity (≥5 deliveries) predicted poor BCSS but only in luminal B-like (HER2 negative) subtype tumors.

Previous studies have suggested that parity is correlated with either favorable or unfavorable breast cancer prognoses (2, 15, 16). In particular, high parity has been associated with poor outcomes for TNBC and luminal subtype tumors (7, 9, 13). The discrepancies between studies may be due to heterogenous patient material, varying subtype definitions or the dependence on retrospective registry-based studies. There has been a lack of prospective research of parity as an independent prognostic factor using modern definitions of the intrinsic subtypes. To the best of our knowledge, the present research is the first single-institution study to use real-world prospective patient data to assess parity as a BCSS prognostic factor.

We used widely recognized ESMO 2015 guidelines for subtyping our breast cancer cases. Thus, five subtypes, based on their ER, PR, Ki-67, and HER2 expression were used: luminal A-like, luminal B-like (HER2 negative), luminal B-like (HER2 positive), TNBC and HER2 positive (non-luminal).

It has been previously reported that parity is differentially associated with breast cancer subtypes—in particular, that parous women are more likely to have TNBC than luminal A breast cancers (22). However, we found that high parity was prognostic of poor outcomes, when tumor size and nodal status were controlled for, but only in luminal B-like (HER2 negative) subtype cancers. This suggests that parity may have biological effects that extend to later diagnoses of breast cancer and its metastasis, particularly in estrogen-dependent, rapidly proliferating breast cancers. On the other hand, it seems that having four or less deliveries is not sufficient to induce such changes. Although it is always difficult to assess causality in a cohort setting, this observed link between higher parity and worse BCSS fulfills most of Bradford Hill's classic causation criteria (23).

Luminal B-like breast cancer subtypes are associated with greater tumor aggressiveness and with significantly worse prognoses than the luminal A-like subtypes (24–26). Despite their positive ER expression, luminal B-like tumors may utilize alternative pathways for growth, since they do not show an equivalent expression of estrogen-regulated genes (24). It has been suggested that ER expression plays an essential role in parity-related changes, since the hormonal milieu of pregnancy changes the mammary microenvironment, thus potentially stimulating growth (27). It has also been speculated that pregnancy may significantly affect ER positive tumor progression (28). It is plausible that the known alternative pathways for growth, such as the epidermal growth factor receptor and PI3K/AKT/mTOR pathways, are responsible for the association between poor prognoses and high parity in luminal B-like (HER2 negative) subtype breast cancers (26).

The hypothesis of postpartum mammary gland involution suggests that changes in the extracellular matrix occur due to activation of collagen remodeling and fibrillar collagen disposition (5, 29). The post-pregnancy mammary microenvironment may thus become cancer-promoting by inducing metastasis through the collagen, COX2 and wound-healing pathways (5, 6). In addition, these metastasis-promoting changes in the breast may be present for only a certain period of time, since only breast cancer diagnoses within 10 years of last childbirth have been shown to be an independent prognostic factor for distant metastasis (30). Moreover, the subtype-specific changes in the extracellular matrix may be related to the relationship between high parity and luminal B-like (HER2 negative) tumors' prognoses. For instance, Bergamaschi et al. (31) studied extracellular matrix gene profiles in breast cancer and were able to divide patients into different prognostic groups based on their tumors' gene expressions. Furthermore, histologically defined breast cancer subtypes were not equally present among the gene expression groups, potentially suggesting that the extracellular matrix differed between subtypes. However, the histological subtypes used by Bergamaschi et al. differed from those used in the present study and are thus not directly comparable.

Overall, we observed excellent long-term outcomes in our prospective cohort, with a 10-year BCSS of 91.4%. The best BCSS was among patients with luminal A-like subtype tumors, among whom only 2.1% died due to breast cancer in our 10-year Kaplan-Meier estimate. This is consistent with results from a population-based study utilizing molecular subtype divisions that found that luminal A-like subtypes were associated with the lowest 10-year all-cause mortality of all tumor subtypes, while the HER2-enriched subtype had the highest (18). Similarly, Hennigs et al. (17) recently reported on a large prospective single-center study assessing the 5-year outcomes of non-metastatic breast cancer patients; they found that patients with luminal A-like subtype (IHC surrogate) tumors had the best prognoses in terms of overall survival (OS), disease-free survival, distant disease-free survival and relative OS. Furthermore, although they assessed patients during a notably shorter follow-up period than we did, they reported 5-year relative OS (BCSS not reported) in the whole cohort to be 94.7%; this is similar to our present finding that 5-year BCSS was 95.8%. Likewise, another retrospective study by Deniz et al. (32) found that luminal A-like subtype cancers had the most favorable outcomes; they observed 5-year BCSS to be 96.5%, compared to our present finding of 99.6%. In addition, both Hennigs et al. and Deniz et al. found that the TNBC subtype had the least favorable 5-year prognoses (OS 78.5% and BCSS 87.6%, respectively) (17, 32). This is consistent with our finding that TNBC subtypes had the worst 5-year prognosis, with a BCSS of 85.6%. Interestingly, however, we found that luminal B-like (HER2 positive) subtype tumors had the worst 10-year BCSS, at 80.6%, while TNBC-subtype tumors had a 10-year of BCSS of 83.9%. However, in all these studies, as well as in the present research, luminal A-like subtype tumors were the most frequently diagnosed (17, 18, 32).

The present study has some limitations. First, we defined the molecular subtypes using IHC molecular subtype surrogates instead of gene expression profiling, potentially leading to some inaccuracies in the data, since IHC relies on a more subjective assessment than gene expression profiling. However, all of our IHC procedures have been validated, thereby reducing this concern. Second, another limitation is that we were unable to gather information on some potentially confounding reproductive factors, such as age at first birth, breastfeeding history, time between last birth and breast cancer onset, and use of oral contraceptives and hormone replacement therapy (15, 33–35). Thirdly, the number of patients and especially the number of breast cancer-related deaths in the patients with luminal B-like (HER2 negative) cancer was rather low, 25. These results should be therefore evaluated cautiously and need to be confirmed in a larger material. Nonetheless, our study has a number of strengths, including its modern definition of breast cancer subtypes according to the ESMO Early Breast Cancer Clinical Practice Guidelines, its assessment of subtype-specific BCSS, its median follow-up time of 8.5 years and its use of contemporary treatment modalities and modern diagnostics in a university hospital. In addition, all of our patient data were reliably collected and stored.

In conclusion, ≥5 deliveries before breast cancer diagnosis predicted poor BCSS but interestingly only in luminal B-like (HER2 negative) subtype tumors. Although we observed excellent overall long-term breast cancer–specific outcomes, there was an absolute difference of 17.3% between the 10-year BCSS of the subtype with the best prognosis (luminal A-like) and the subtype with the worst prognosis (luminal B-like HER2 positive). These subtype-specific 10-year BCSS results provide essential additional data about long-term outcomes using modern treatment modalities. However, further research is needed to establish the prognostic associations between parity and individual molecular breast cancer subtypes.

## Data Availability Statement

The datasets generated for this study are available on request to the corresponding author.

## Ethics Statement

The study was approved by the Local Ethics Committee of the Ostrobothnia Hospital District (114/2011) and the National Supervisory Authority for Welfare and Health (D9580/05.01.00.06/2010). All studies were conducted in accordance with the principles of the Declaration of Helsinki and the guidelines for good clinical practice. The patients provided their written informed consent to participate the study. The data that support the findings of this study are available from the corresponding author upon reasonable request.

## Author Contributions

AJ, NR, and PK collected and analyzed the study material. All the authors participated in writing the article and have contributed significantly to the work, read the manuscript, attested to the validity and legitimacy of the data and its interpretation, and agreed to its submission to the Frontier in Oncology.

## Conflict of Interest

The authors declare that the research was conducted in the absence of any commercial or financial relationships that could be construed as a potential conflict of interest.
